# Thermal limits for flight activity of field-collected *Culicoides* in the United Kingdom defined under laboratory conditions

**DOI:** 10.1186/s13071-020-04552-x

**Published:** 2021-01-18

**Authors:** Laura A. Tugwell, Marion E. England, Simon Gubbins, Christopher J. Sanders, Jessica E. Stokes, Joanne Stoner, Simon P. Graham, Alison Blackwell, Karin E. Darpel, Simon Carpenter

**Affiliations:** 1 The Pirbright Institute, Ash Road, Woking, GU24 0NF UK; 2 School of Veterinary Medicine, University of Surrey, Daphne Jackson Rd, Guildford, GU2 7AL UK; 3 APS Biocontrol Ltd, Prospect Business Centre, Dundee, DD2 1TY UK

**Keywords:** Ceratopogonidae, *Culicoides*, Bluetongue virus, African horse sickness virus, Phototaxis, Thermal limits

## Abstract

**Background:**

*Culicoides* biting midges (Diptera: Ceratopogonidae) are biological vectors of internationally important arboviruses and inflict biting nuisance on humans, companion animals and livestock. In temperate regions, transmission of arboviruses is limited by temperature thresholds, in both replication and dissemination of arboviruses within the vector and in the flight activity of adult *Culicoides*. This study aims to determine the cold-temperature thresholds for flight activity of *Culicoides* from the UK under laboratory conditions.

**Methods:**

Over 18,000 *Culicoides* adults were collected from the field using 4 W down-draught miniature ultraviolet Centers for Disease Control traps. Populations of *Culicoides* were sampled at three different geographical locations within the UK during the summer months and again in the autumn at one geographical location. Activity at constant temperatures was assessed using a bioassay that detected movement of adult *Culicoides* towards an ultraviolet light source over a 24-h period.

**Results:**

The proportion of active adult *Culicoides* increased with temperature but cold temperature thresholds for activity varied significantly according to collection season and location. Populations dominated by the subgenus *Avaritia* collected in South East England had a lower activity threshold temperature in the autumn (4 °C) compared with populations collected in the summer (10 °C). Within the subgenus *Avaritia*, *Culicoides scoticus* was significantly more active across all temperatures tested than *Culicoides obsoletus* within the experimental setup. Populations of *Culicoides impunctatus* collected in the North East of England were only active once temperatures reached 14 °C. Preliminary data suggested flight activity of the subgenus *Avaritia* does not differ between populations in South East England and those in the Scottish Borders.

**Conclusions:**

These findings demonstrate seasonal changes in temperature thresholds for flight and across different populations of *Culicoides.* These data, alongside that defining thresholds for virus replication within *Culicoides,* provide a primary tool for risk assessment of arbovirus transmission in temperate regions. In addition, the study also provides a comparison with thermal limits derived directly from light-suction trapping data, which is currently used as the main method to define adult *Culicoides* activity during surveillance. 
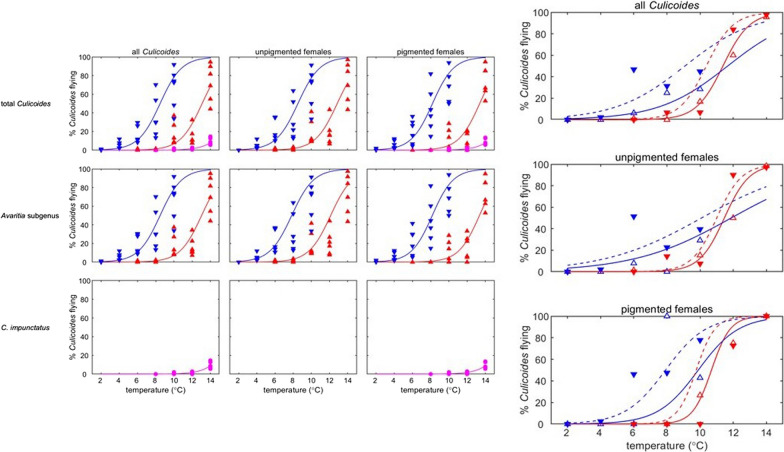

## Background

*Culicoides* biting midges (Diptera: Ceratopogonidae) are biological vectors of a range of internationally important arboviruses of companion animals, livestock and wild mammals, including bluetongue virus (BTV), African horse sickness virus (AHSV) and Schmallenberg virus (SBV) [1, 2]. In addition, *Culicoides* transmit Oropouche virus (OROV), which causes a febrile illness in humans, and play a poorly defined role in the transmission of a range of other zoonotic arboviruses [3, 4]. Within northern Europe, both BTV and SBV have a significant impact on livestock production through clinical disease and also ruminant movement controls and trade restrictions imposed to reduce virus spread [5, 6]. The risk of major shifts in the global distribution of *Culicoides*-borne arboviruses is currently considered to be high [7, 8], as illustrated by the recent unprecedented emergence of AHSV in Thailand [9].

In temperate regions, such as north-western Europe, temperature has a fundamental influence on the transmission and persistence of *Culicoides*-borne arboviruses [10]. The rate of arbovirus replication and dissemination within a biological vector is usually highly dependent on environmental temperature, as most arthropods are poikilothermic. In the case of *Culicoides*-BTV interactions, the threshold for infection and replication lies within a range of 11–15 °C [11]. It is clear, however, that the temperature threshold for *Culicoides* active flight is below this range and therefore could allow survival of the virus through winter periods when replication does not occur, but when infected adults are able to survive, fly and feed on hosts [12, 13]. It has also been demonstrated that *Culicoides* infected with AHSV at 25 °C and then immediately held at a constant temperature of 10 °C for 35 days develop full infections following a return to 25 °C for 3 days [14]. Hence, resumption of temperatures conducive to virus replication could also lead to transmission during transient warmer periods within winter.

Temperature-related thresholds to activity in adult *Culicoides* can be assessed through direct analysis in the laboratory and through a variety of field-based methods. Direct studies of activity use laboratory-based bioassays based on a phototactic response while they are held at a known temperature. A recent study in the Republic of South Africa (RSA) examined the major afrotropical vector of arboviruses, *Culicoides* *imicola* Kieffer, and measured flight response using a white fluorescent light stimulus and horizontal flight over a distance of approximately 15 cm [15]. An earlier study, based in Japan, examined activity using a dedicated bioassay system based on a requirement for 8 cm of horizontal flight in response to an ultraviolet light source [16]. In both *Culicoides oxystoma* Kieffer and *Culicoides* *maculatus* (Shiraki), active flight was recorded in < 10% of individuals at temperatures < 16–17 °C, although a few individuals were recorded as being active even at the minimum temperature of 6 °C. In contrast to the study in RSA, over 80% of individuals exhibited a phototactic response at temperatures of greater than 25 °C.

Activity of adult *Culicoides* can also be measured in the field by collection in parallel with measurement of environmental temperature. The most epidemiologically relevant method of assessing thermal limits to activity is to measure adult *Culicoides* blood-feeding behaviour on a relevant host. A key issue is that these studies are generally carried out at times of peak adult seasonal abundance, where thermal limits to activity are not usually reached, and are also limited by the availability of daylight [17]. As an example, collections of adult *Culicoides* from sheep made in the UK using a drop-trap design reported all species within the subgenus *Avaritia* as active across the entire temperature sampling range of 11.8–29.0 °C between the daylight hours of 18:00 and 21:30 [17]. Truck trapping has also been carried out in the UK using a vehicle driven over a set course with a net mounted on its roof [18]. This samples populations of adult *Culicoides* actively flying at a set height of approximately 1.5 m without an attractant. *Culicoides* of the subgenus *Avaritia* were collected over a range of 8.6–30.0 °C, with larger catches associated with higher temperatures, although reductions in activity were recorded when the temperature exceeded 21 °C [18]. Both of these approaches are usually only deployed at single sites on a local scale due to their complex logistics.

Thermal limits to *Culicoides* adult activity can be loosely inferred from less labour-intensive trapping approaches such as, ultraviolet (UV) light-suction traps. Despite their widespread use in Europe for both research activities and surveillance of *Culicoides* during arbovirus outbreaks [19–21], data collected from winter periods are limited, making it difficult to examine cold temperature thresholds for activity. In France, adult *Culicoides* activity was inhibited, as measured by an absence of individuals in trap collections, on nights when the maximum temperature fell below 10 °C [22], and a similar result was found in Germany where activity was limited to days with a maximum temperature of ≥ 10.8 °C [23]. A field study in the UK was able to collect adult *Culicoides*, using UV light-suction traps, in February and March when the maximum temperature for both months was 10.1 °C [24]. Results from a large dataset of *Culicoides* collections across Europe indicate that the onset of the vector season can occur at mean temperatures as low as 1 °C in the most northern latitude tested, whereas mean temperatures of 10 °C are required for *Culicoides* activity in countries in the southern latitudes [21]. Key variations in experimental methods include the method by which temperature is inferred (e.g. *in situ*, using national weather station-based monitoring or remotely sensed data) and the fact that light-suction trap collections are not representative of biting rate on hosts in this region [17].

Surveillance of adult *Culicoides* flight periods, through the use of light-suction traps, is currently adopted in Europe to determine the time period during which the probability of arbovirus transmission is very low, defined as the seasonal vector-free period (SVFP) [25]. Understanding the temperature threshold for flight activity in *Culicoides* is also critical in evaluating the risk of transmission following an incursion, in addition to understanding their role in arbovirus overwintering in the UK [12]. The implementation of trap surveillance, if not already in place, is time-consuming resulting in a delay of active surveillance from the point of initial incursion. Applying easily accessible temperature data, however, to evaluate the activity rates of *Culicoides* populations and thus infer the risk of transmission by active *Culicoides* could overcome the issues associated with post-incursion surveillance. The aim of this study was therefore to determine a cold temperature threshold for flight activity under experimental conditions for field-caught UK *Culicoides* species, collected at different seasons and geographical locations.

## Methods

### Study sites

**Fig. 1:**
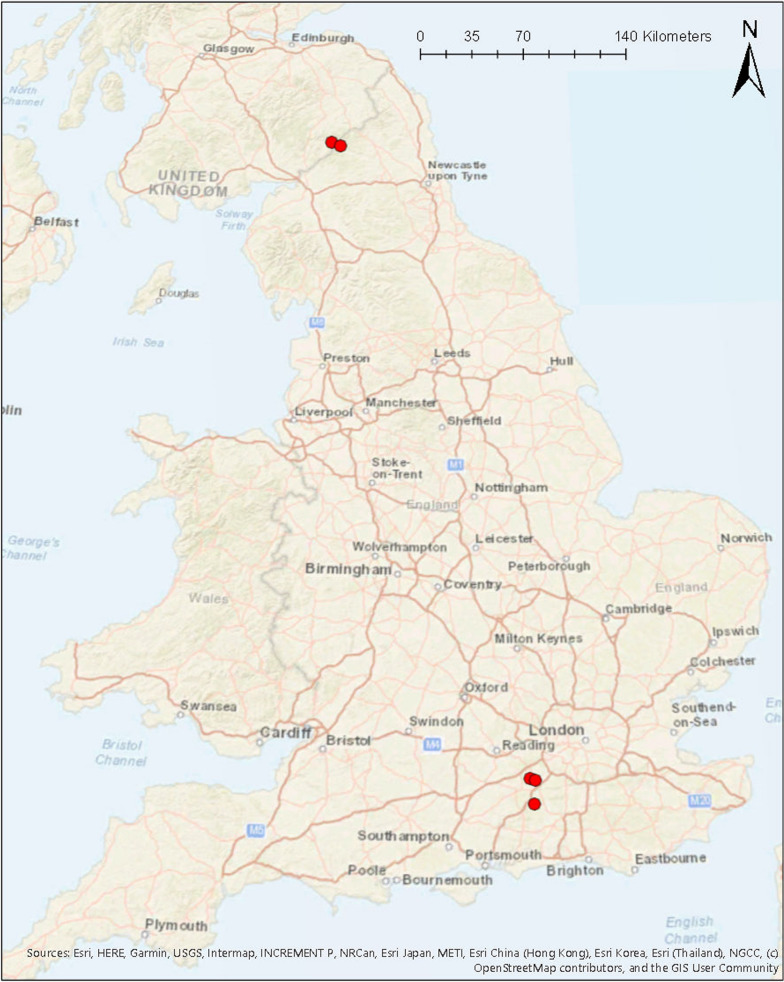
Map of study sites. A total of five study sites were used: sites 1, 2 and 3 in South East England, site 4 in North East England and site 5 in the Scottish Borders. The red points on the map indicate each study site from where *Culicoides* were collected

Field collections of adult *Culicoides* were conducted across five sites in the UK (Fig. 1) at different times of the year (Additional file 1: Table S1). In South East England, three equine holdings (site 1: 51°08′46.5″N, 0°36′51.6″W; site 2: 51°17′26.2″N, 0°39′09.0″W; site 3: 51°17′00.3″N, 0°36′24.6″W) were used for collections in June–August 2017 (South East England summer cohort; SES) and again for collections in September–October 2017 (South East England autumn cohort; SEA). All three sites were predominately used for grazing, with animal stabling and muck heaps present at each site. In North East England, collections were made from a forest campsite (site 4: 55°14′13.1″N, 2°35′12.1″W) during July 2018 (North East England summer; NES). During the same month, a collection was also made at a mixed farm in the Scottish Borders (site 5: 55°15′44.5″N, 2°41′16.4″W) (Scottish Borders summer cohort; SBS).

### Collection methods

Insects were collected using down-draught miniature blacklight (UV) Centers for Disease Control (CDC), model 912 traps (John W Hock Co, Gainesville, FL, USA). Each CDC trap uses a 4 W UV tube and is powered by a 12 V lead acid sealed battery (Yuasa, Japan). At each site, between four and eight traps were used to catch sufficient *Culicoides* for the trials and were positioned at least 50 m apart [26]. Traps were suspended at a height of approximately 1.5 m above ground. Traps were set up at least 2 h before sunset and run overnight before collection within 2 h of sunrise the following day. Insects were collected live into a 340 ml cardboard collection cup containing a cotton pad soaked in 10% sucrose to provide a sugar source and paper towel cut into long, thin strips to provide shelter from the trap fan downdraft. Cardboard collection cups were secured to the CDC trap using a CDC sleeve and elastic bands (Additional file 2: Figure S1). The following morning, sleeves from each trap were removed and tied with a clip-lock tie to contain the insects within the collection cup and sleeve.

### Flight activity experiments

**Fig. 2:**
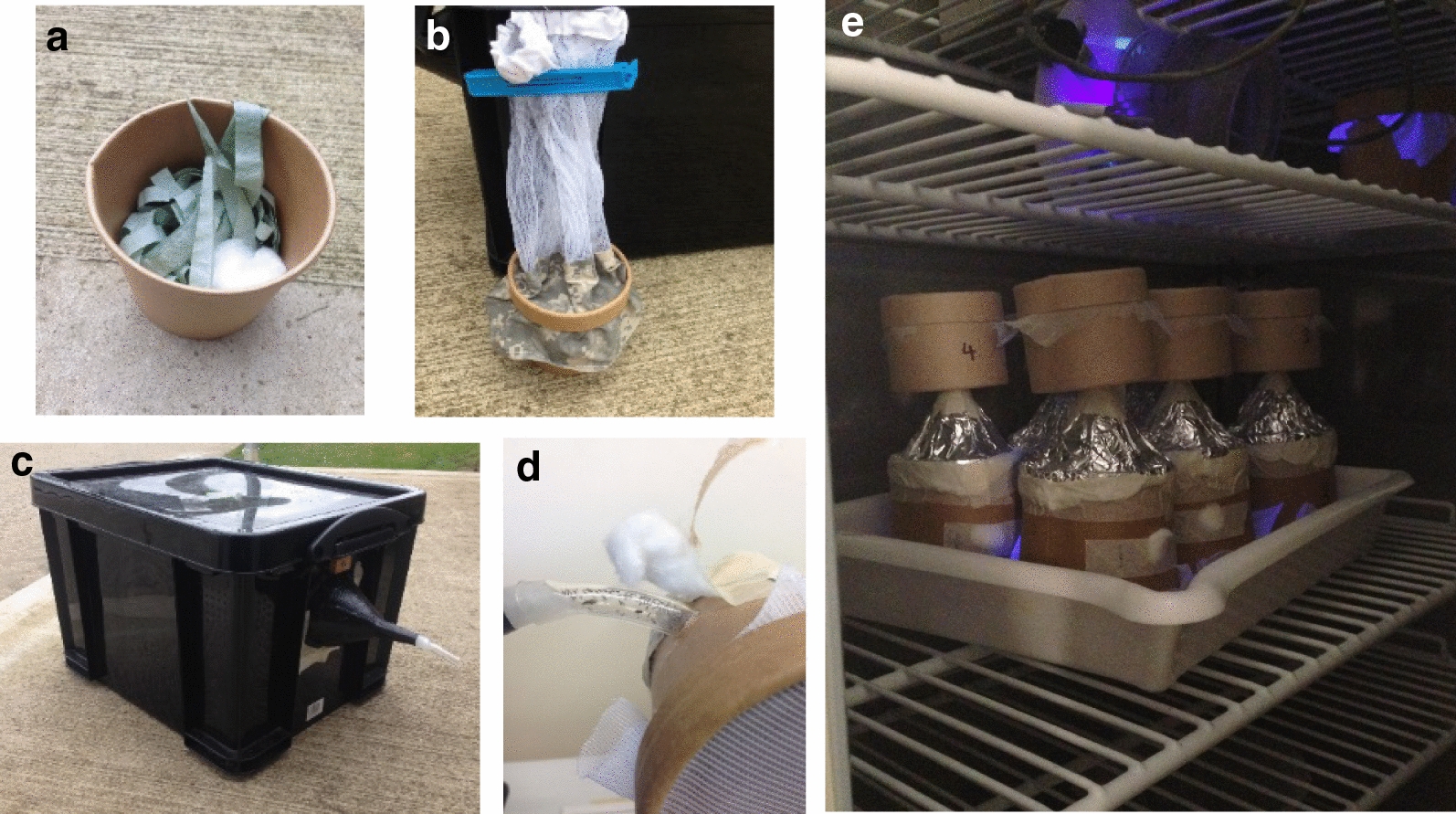
Photographs of equipment used for collecting and sorting *Culicoides* collections for the flight activity study. Cardboard collection cup (340 ml) containing a sucrose-soaked cotton wool pad and paper towel strips (**a**); collection cup and sleeve removed from trap and secured using clip-lock tie (**b**); black plastic box with funnel at one end with attached clear plastic tubing (**c**); insects entering flight activity pots from the dark plastic box via the funnel and clear tubing (**d**); flight activity pots 1–6 were transferred into dark incubators and specifically were placed with the mesh downwards onto a tray to ensure darkness within the flight activity pot. A small sucrose-soaked cotton wool pad was placed underneath and a 7.5-cm-diameter pill box, with a fine mesh lid, was secured on top of the funnel allowing active insects to fly towards the UV light stimulus into the attached pill box (**e**)

Insects collected were transferred and released into a large plastic, black box (42 cm [l] × 35 cm [w] × 32 cm [h]) with a translucent funnel (14 cm long) attached on one side to allow for insects to exit. Clear plastic tubing (6 cm in length) was secured to the translucent funnel to visualise and count approximately 150–250 active, phototactic adult *Culicoides* exiting the box and entering a ‘flight activity pot’ (Fig. 2). The flight activity pots were modified 340 ml cardboard collection cups with a small plastic funnel (66 mm top diameter; 7.4 cm in length) covered in aluminium foil fitted at the top and a fine mesh covering the bottom of the pot. The total height of the flight activity pot from the base of the cardboard cup to the tip of the funnel was 14 cm. A cotton wool bung within the funnel retained all insects within the flight activity pot until the start of the experiments.

Once eight flight activity pots had been filled, they were numbered at random from one to eight. Pots one to six were placed into a ‘test’ temperature-controlled incubator (MIR-254 Panasonic, UK) at an initial temperature of 20 ± 1 °C, while pots seven and eight were placed in a second ‘maintenance’ incubator also set at 20 ± 1 °C as a control for activity of *Culicoides* in the sample. Both incubators contained a UV light source (4 W blue-blacklight tube; John W Hock Co, Gainesville, FL, USA) suspended within the incubator that acted as an attractant and no other light was provided within the incubators. The test incubator was then set to the desired temperature (2 °C–14 °C) and insects were allowed to acclimatise for 2 h. Following the acclimatisation period, the cotton wool bung at the top of the flight activity pot funnel was removed and a 7.5-cm-diameter pill box (Watkins and Doncaster, UK) with a fine mesh lid was secured on top of the funnel allowing active insects to fly towards the UV light stimulus into the attached pill box (Fig. 2e). All flight activity pots were placed with the mesh lid faced downwards onto a tray to ensure darkness, so that the UV light was the only light source available above the flight activity pots. A small cotton pad soaked in 10% sucrose was placed underneath each flight activity pot on the tray, rather than within the pot, so that insects could still access the sugar-meal through the fine mesh but were prevented from sticking to the pad.

At regular intervals the collection pill boxes were replaced (0.5-, 1-, 2-, 4-, 6-, 8-, 10- and 24-h intervals) and the collected *Culicoides* were killed by freezing at − 20 °C for a minimum of 2 h. All remaining inactive individuals in the flight activity pots were also killed by freezing after 24 h. The percentage of *Culicoides* flying after 24 h was determined by calculating the mean cumulative proportion of active *Culicoides* across the six flight activity pots maintained at each test temperature. In each temperature trial, collections were also made at the same intervals from pots seven and eight, which were maintained at 20 ± 1 °C throughout the experiment to ensure the population tested were active without the constraint of cold temperatures. A flow diagram of the full experimental design is provided in Additional file 3: Figure S2. A range of temperatures was tested for the SES, SEA and NES cohorts to cover a range of activity levels that would allow for the determination of a cold temperature threshold. The temperature ranges were ascertained after measuring *Culicoides* flight activity levels at an initial test temperature of 10 °C. In the SES cohort, five temperatures were tested (6, 8, 10, 12 and 14 °C) and the SEA cohort a further five temperatures were tested (2, 4, 6, 8 and 10 °C). Four temperatures were tested in the NES cohort (8, 10, 12 and 14 °C) and due to time restrictions only one temperature of 12 °C was tested in the SBS cohort.

### Sample identification

Only individuals that were tested in activity experiments were identified; all surplus insects that remained in the initial black plastic sorting box were not used for experimental study. *Culicoides* from the activity experiments were sorted morphologically under a dissecting microscope using characteristic wing patterns with the aid of an identification key [27]. Adult *Culicoides* were grouped into six categories: subgenus *Avaritia*, *Culicoides* *pulicaris* (Linnaeus)*, Culicoides punctatus* (Meigen)*, Culicoides achrayi* Kettle and Lawson*, C. impunctatus* and other *Culicoides*. Female *Culicoides* were also identified to physiological state by examination of the abdomen [28] and assigned to one of the following categories: unpigmented, pigmented, gravid and blood-fed.

For both South East England cohorts (SES and SEA), a sub-sample of females within the subgenus *Avaritia* was identified further to species level (*C. obsoletus* and *C. scoticus* only) using an adapted multiplex polymerase chain reaction (PCR) method targeting the internal transcribed spacer (ITS) 1-5.8S-ITS2 region [29]. All individuals belonging to the subgenus *Avaritia* from one flight pot from each temperature trial were chosen at random for molecular analysis and used as a representative sample for each population at each temperature trial. *Culicoides* were transferred to individual reaction tubes with 200 µl of tissue digest solution containing 100 nM Tris–HCl pH8 (Thermo Fisher, UK), 200 mM NaCl (Sigma-Aldrich, UK), 0.2%(w/v) SDS (Thermo Fisher), 5 mM EDTA (Thermo Fisher), 200 µg/ml proteinase K (Thermo Fisher) and nuclease-free water (Thermo Fisher). Following an overnight incubation in tissue digest solution at 37 °C, individual *Culicoides* specimens were transferred to tubes containing 70% ethanol for storage. The *Culicoides* DNA was then extracted from 100 µl of tissue digest solution and eluted into 100 µl buffer using the KingFisher Flex automated extraction platform and the MagMAX™ CORE Nucleic Acid Purification Kit (Thermo Fisher) according to the manufacturer’s instructions.

Two microlitres of each sample DNA was added to each well on a PCR plate (Life Technologies, UK), each containing 8 µl of mastermix, which consisted of 1 × TaqMan Fast Advanced MasterMix (Thermo Fisher), 0.3 µM of each primer [29] and 0.2 µM of each probe [29], and diluted to a total reaction volume of 10 µl using nuclease-free water (Thermo Fisher). Negative extraction controls consisted of elution from wells which did not contain any *Culicoides* specimen in the extraction plate and negative PCR controls contained just nuclease-free water. At least three negative controls and at least three positive controls, using DNA extracted from males from the same study morphologically identified as either *C. obsoletus* or *C.* *scoticus*, were added to each plate.

The PCR thermal profile used consisted of 2 min at 50 °C for activation of uracil-DNA-glycosylases (UDG), an initial denaturation step of 2 min at 95 °C, followed by 40 cycles of 95 °C for 3 s and 60 °C for 30 s and was carried out using an Applied Biosystems 7500 Fast instrument (Thermo Fisher). Each plate was analysed using the ViiA7 Real-Time PCR system software (Thermo Fisher). Determination of species for each individual specimen was based on the cycle threshold (C_t_) value for each species-specific primer-probe pairing. Negative samples were defined as having a C_t_ ≥ 35 and positive samples were defined as having a C_t_ ≤ 25. Samples with a C_t_ between > 25 and < 35 were regarded as undetermined and were repeated. If samples remained undetermined following re-examination, samples were defined as unknown and were removed from analysis.

### Statistical modelling

Generalised linear mixed models (GLMMs) were used to investigate the relationship between temperature and the proportion of *Culicoides* flying and how this relationship differed amongst cohorts. Specifically, a binomial family GLMM with a logit link function was constructed with the proportion of *Culicoides* flying as the response variable. Model selection proceeded by stepwise deletion of non-significant (*P *> 0.05) terms (as judged by likelihood ratio tests), starting from a model including temperature (°C), cohort and an interaction between them as fixed effects and pot as a random effect (to allow for between-pot variation). The models were implemented using the lme4 package [30] in R (version 3.6.1) [31].

Separate models were constructed for total *Culicoides* (all *Culicoides*, unpigmented females and pigmented females), the *Avaritia* subgenus (all *Culicoides*, unpigmented females and pigmented females) and *C. impunctatus* (all *Culicoides* and pigmented females). Sample sizes were insufficient to examine relationships for: (i) *C. pulicaris*, *C. achrayi* or other *Culicoides* or (ii) blood-fed females, gravid females or males for any species/groups. In addition, the SBS cohort was excluded from this analysis as activity was only assessed at a single temperature (12 °C) for this cohort.

Further models were constructed for *Culicoides* flight activity at 12 °C to compare activity amongst the SES cohort, NES cohort and SBS cohort. A similar approach to that described above was used, except the model included only cohort as a fixed effect and pot as a random effect. Flight activity in the cohorts was compared using Tukey multiple comparisons.

For the SES and SEA cohorts, there was a sufficient number of *Culicoides* caught to allow two further analyses. First, to compare flight activity of unpigmented and pigmented females of the *Avaritia* subgenus a GLMM was constructed including pigmentation state (i.e. unpigmented or pigmented) as a fixed effect as well as two- and three-way interactions between it and the other fixed effects (i.e. temperature and cohort). Model selection was carried out as described above. Second, to compare flight activity of *C. obsoletus* and *C. scoticus*, a binomial family generalised linear model (GLM) with a logit link function was constructed. The proportion of *Culicoides* flying was the response variable and temperature, cohort and species were fixed effects. Model selection proceeded as described above.

## Results

For both the SES and SEA cohorts, a total of five collections were made to test five temperatures in each cohort. Each collection, made across multiple sites (sites 1 to 3), was used to test one temperature at a time. In the SES cohort, a total of 5586 *Culicoides* were collected across the five collections to test five temperatures (6, 8, 10, 12 and 14 °C). In the SEA cohort, a total of 5309 *Culicoides* were collected across the five collections to test five temperatures (2, 4, 6, 8 and 10 °C). A total of 7228 *Culicoides* were tested in the NES cohort from a total of four collections from site 4 only to test four temperatures (8, 10, 12, 14 °C). One collection was made in the SBS cohort to test one temperature (12 °C) in which 585 *Culicoides* were tested (Additional file 4: Table S2).

In the South East of England, > 97% of *Culicoides* in both the summer (5463 of the 5586 individuals) and autumn (5259 of the 5309 individuals) cohorts belonged to the subgenus *Avaritia.* In contrast, > 99% of *Culicoides* in North East England were identified as *C.* *impunctatus* (6611 of the 6643 individuals). There were insufficient numbers collected of other species (*C. pulicaris, C.* *punctatus, C. achrayi* and other *Culicoides*) from all cohorts for further analyses to be conducted on these species. (Additional file 4: Table S2). Collections were also dominated by unpigmented and pigmented female *Culicoides* and no further analysis was conducted on males, gravid or blood fed females (Additional file 4: Table S2).

**Fig. 3:**
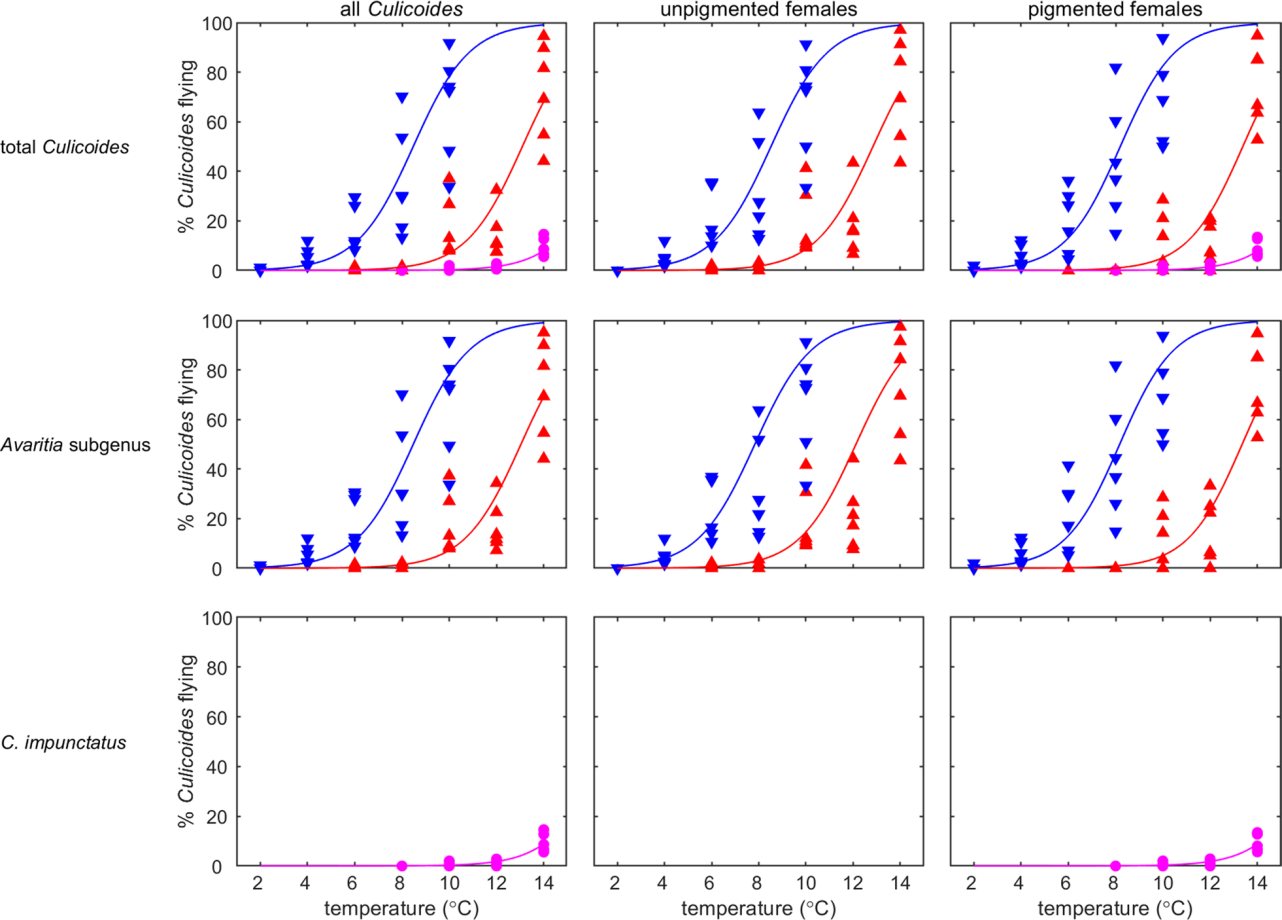
Percentage of *Culicoides* collected in the flight pill box after 24 h at defined temperatures. Rows show results for total *Culicoides* (top row), the *Avaritia* subgenus (middle row) and *Culicoides impunctatus* (bottom row). Columns show results for all *Culicoides* (left), unpigmented females (centre) and pigmented females (right). Symbols show the observed percentage of *Culicoides* flying and the curve is the fitted model (using the estimated fixed effects only). Cohorts are indicated by the curve/symbol colour: SES (red); SEA (blue); NES (magenta). If data are not shown for a cohort, insufficient specimens of that species/group were caught for it to be included in the analysis

In all cohorts where multiple temperatures were tested, the proportion of active *Culicoides* increased as temperature increased within the range tested (Fig. 3; Additional file 5: Table S3). This relationship was observed for total *Culicoides* (all adults, unpigmented females and pigmented females), the subgenus *Avaritia* (all adults, unpigmented females and pigmented females) and *C.* *impunctatus* (all adults and pigmented females) in both South East England cohorts (SES and SEA) and the NES cohort (Fig. 3). Moreover, the rate at which *Culicoides* activity increased with temperature was the same amongst the three cohorts tested, i.e. there was no significant interaction between temperature and cohort (likelihood ratio tests: total *Culicoides* [all, *χ*^2^ = 1.31, df = 2, *P *= 0.52; unpigmented, *χ*^2^ = 0.72, df = 1, *P *= 0.40; unpigmented, *χ*^2^ = 3.70, df = 2, *P *= 0.15] and subgenus *Avaritia* [all, *χ*^2^ = 0.86, df = 1, *P *= 0.35; unpigmented, *χ*^2^ = 0.80, df = 1, *P *= 0.37; unpigmented, *χ*^2^ = 3.01, df = 1, *P *= 0.08]).

The season and geographical location of *Culicoides* collection influenced the temperature at which *Culicoides* activity began and the levels of activity reached at each temperature (Fig. 3, Additional file 5: Table S3). Populations collected from North East England (NES cohort), which primarily comprised *C.* *impunctatus*, had a cold temperature threshold for activity, as measured by the minimum temperature at which > 5% of the *Culicoides* population is active, of 14 °C (Fig. 3), whereas the two collections made in South East England (SES and SEA cohorts), which were dominated by *Culicoides* of the *Avaritia* subgenus, had substantially higher activity rates at all temperatures compared to *Culicoides* collected in the NES cohort (Fig. 3). Yet, the cold temperature threshold for activity was lower for adult *Culicoides* in the SEA cohort (4 °C) compared with those collected in the SES cohort (10 °C), though the two populations demonstrated similar activity levels at 14 °C (Fig. 3). This pattern was similar for both the unpigmented and pigmented females in these cohorts (Fig. 3). Indeed, there were no significant (likelihood ratio test, *χ*^2^ = 0.19, df = 1, *P *= 0.66) differences in flight activity between unpigmented and pigmented females of the *Avaritia* subgenus for both the SES and SEA cohorts. In all temperature trials, *Culicoides* from the same populations maintained at 20 ± 1 °C within control pots were active, with average activity levels in control pots of 86% and 78% for the SES and SEA cohorts, respectively, indicating that any reduction in activity is a result of the colder temperatures in temperature trial pots.

A single collection was subsequently made at a site in the Scottish Borders in the summer (SBS) which consisted of a greater proportion of livestock-associated species of *Culicoides* compared with collections made in the NES cohort, collected less than 8 km away; a total of 210 of the 585 individuals tested from the SBS cohort were classified in the subgenus *Avaritia* whereas only 16 of the 6643 individuals tested from the NES cohort were classified in the subgenus *Avaritia*.

**Fig. 4:**
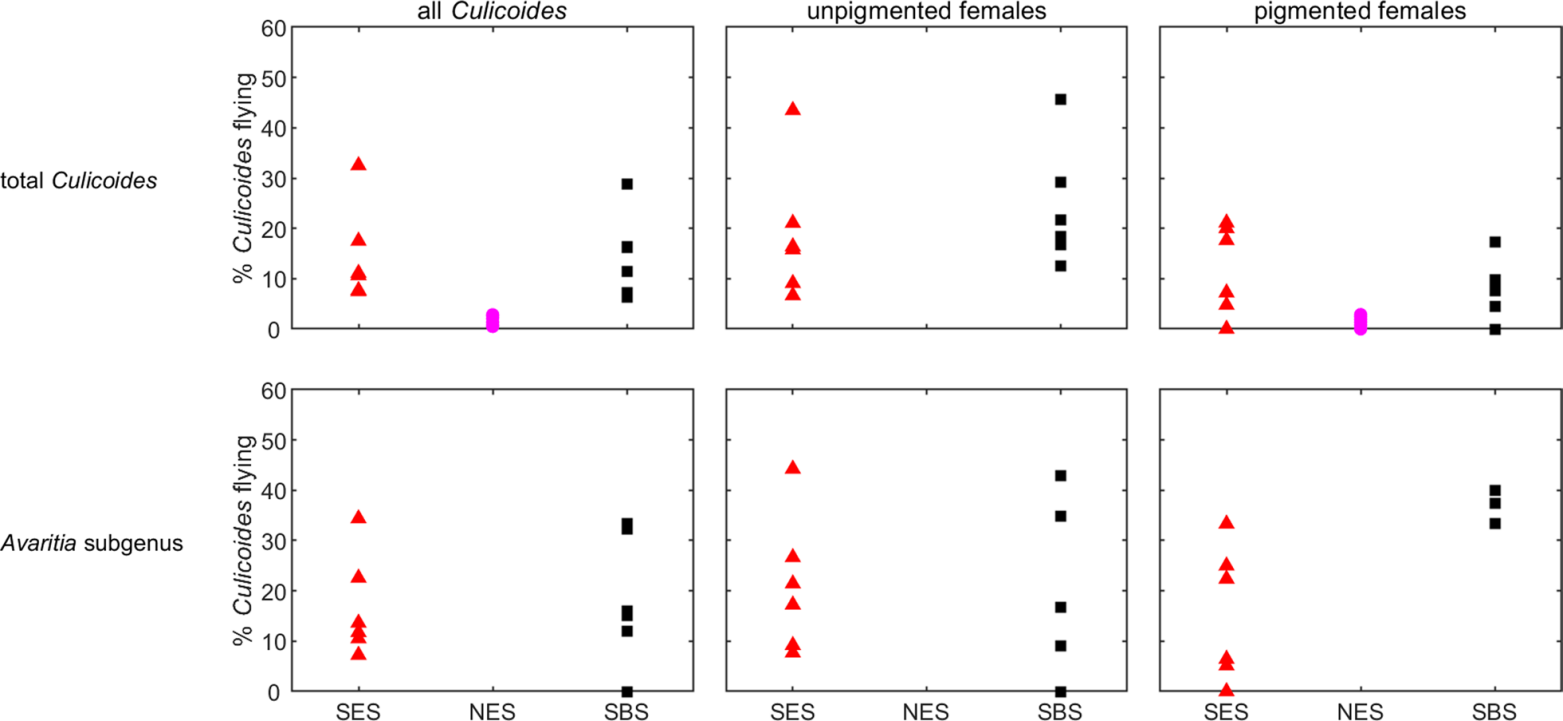
Percentage of *Culicoides* collected in the flight pill box after 24 h at 12 °C. Rows show results for total *Culicoides* (top row) and the *Avaritia* subgenus (bottom row). Columns show results for all *Culicoides* (left), unpigmented females (centre) and pigmented females (right). Symbols show the observed percentage of *Culicoides* flying, with cohorts indicated by the symbol colour: SES (red); NES (magenta); SBS (black). If data are not shown for a cohort, insufficient specimens of that species/group were caught for it to be included in the analysis

Adult *Culicoides* collected in the SBS cohort had a significantly greater flight activity at 12 °C, the only temperature tested, compared with *Culicoides* collected nearby in the NES cohort, when measuring total *Culicoides* and pigmented females (Tukey post hoc comparisons: total, *Z *= 6.09, *P *< 0.001; pigmented females: *Z *= 4.84, *P *< 0.001). Flight activity for *C. impunctatus* specifically at 12 °C, however, was not significantly different between the NES and SBS cohorts (Tukey post hoc comparison, *Z *= 6.09, *P *= 0.34). Flight activity at 12 °C was not significantly different between the SES cohort and the SBS cohort for total *Culicoides* (Tukey post hoc comparison, *Z *= 0.08, *P *= 0.99), total unpigmented females (Tukey post hoc comparison, *Z *= 0.79, *P *= 0.43) and total pigmented females (Tukey post hoc comparison, *Z *= -0.76, *P *= 0.73) (Fig. 4, Additional file 6: Table S4). For adults belonging to the subgenus *Avaritia* specifically, flight activity at 12 °C was higher for the SBS cohort compared with the SES cohort, for pigmented females only (Tukey post hoc comparison, *Z *= 2.71, *P *= 0.007). For all other physiological states within the subgenus *Avaritia*, activity levels between the SES and SBS cohorts were not significantly different (Tukey post hoc comparisons: total, *Z *= 0.82, *P *= 0.42; unpigmented, *Z *= 0.19, *P *= 0.85).

**Fig. 5:**
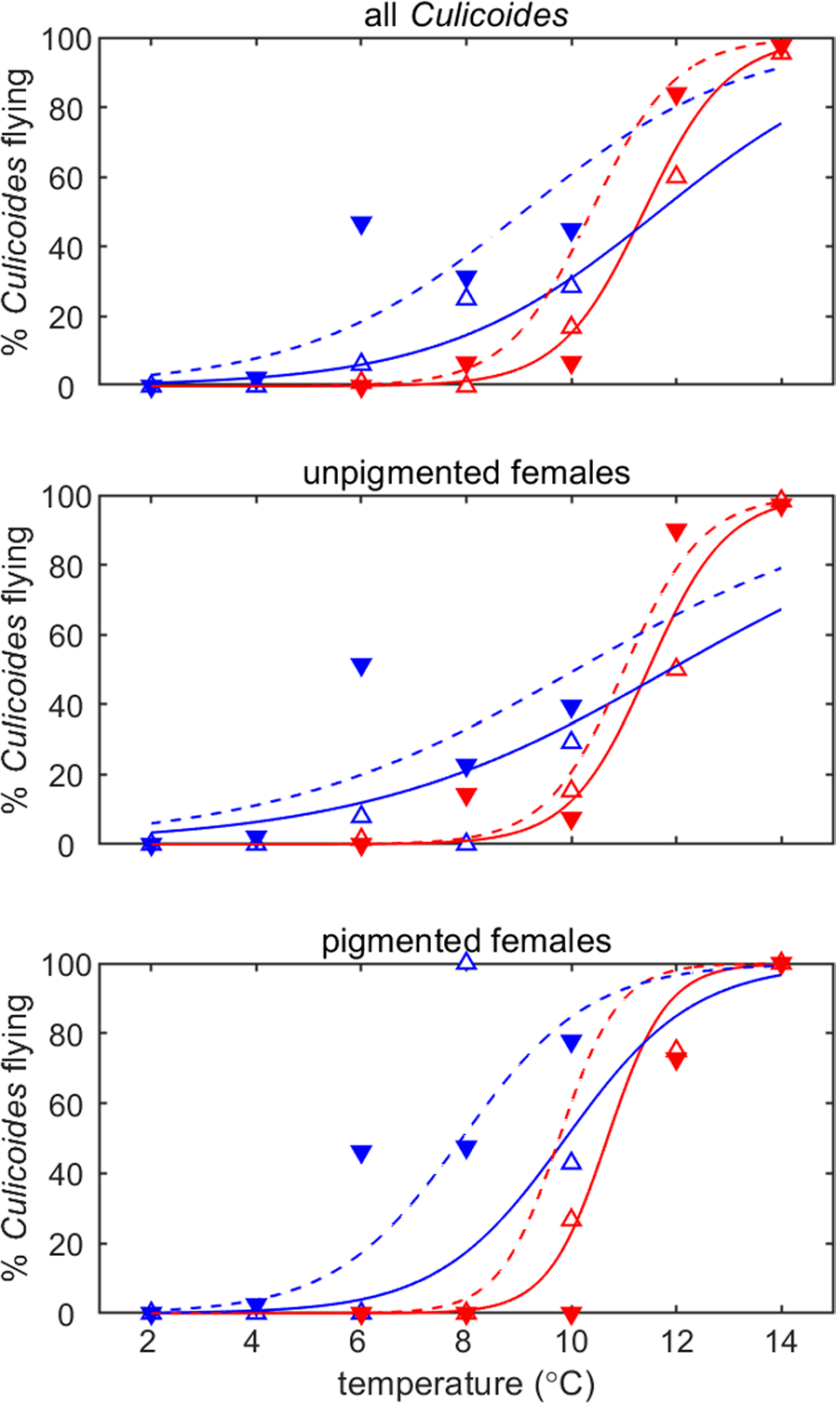
Percentage of *Culicoides obsoletus* and *Culicoides scoticus* collected in the flight pill box after 24 h. Flight activity is shown for total *Culicoides* (top row), unpigmented females (middle row) and pigmented females (bottom row). Symbols show the observed percentage of midges flying and the curve is the fitted model for *C. obsoletus* (open triangles and solid lines) and *C. scoticus* (filled triangles and dashed lines). Cohorts are indicated by the curve/symbol colour: SES (red) and SEA (blue)

Within the South East of England, the two cryptic species within the subgenus *Avaritia, C.* *obsoletus* and *C. scoticus* differed in their flight activity (likelihood ratio tests: total, *χ*^2^ = 4.71, df = 1, *P *= 0.03; unpigmented, *χ*^2^ = 5.47, df = 1, *P *= 0.02; unpigmented, *χ*^2^ = 5.54, df = 1, *P *= 0.02), with a greater proportion of *C.* *scoticus* than *C. obsoletus* active at all temperatures (Fig. 5, Additional file 7: Table S5). As seen in the previous analyses, adults of each species from the SEA cohort were more active at lower temperatures than those from the SES cohort (Fig. 5). Additionally, there was a greater number of individuals from the subgenus *Avaritia* subsample identified as *C. scoticus* later in the season in the SEA cohort (84%) when compared to collections made earlier in the season in the SES cohort (37%).

## Discussion

This study has identified significant differences in temperature thresholds for flight activity of adult *Culicoides* according to species and season under laboratory conditions. In the populations examined in South East England, which were dominated by *Culicoides* of the subgenus *Avaritia*, the cold temperature activity threshold was higher for populations tested earlier in the season (SES: 10 °C) compared to those collected later in the season (SEA: 4 °C), despite similar activity rates being recorded at higher temperatures. In addition, species-specific differences in activity were recorded at an interspecific level with greater activity being recorded across the temperatures tested for *C. scoticus* relative to *C.* *obsoletus*. In addition, *C. impunctatus* collected in North East England (NES: 14 °C) demonstrated significantly reduced activity across all temperatures despite an identical process of acclimatisation and test of flight fitness prior to use in the bioassay.

Studies carried out in South East England utilised two separate populations taken from summer (June–August) and autumn (September–October). The number of generations occurring each year in *Culicoides* in Northern Europe remains unclear, although bi- or trivoltinism has been suggested in populations of *C. impunctatus* [32] and the subgenus *Avaritia* [33]. This study highlights a significant difference in thermal tolerance of populations sampled in the same year and site using identical methods of selection, acclimatisation and measurement of activity. The underlying mechanism enabling this adaptation is unknown, although previous studies carried out in the USA demonstrated rapid cold hardening in adult *C. sonorensis* through short-term exposure to temperatures of < 5 °C [34]. This study highlights the lack of knowledge that currently exists in the drivers of differences in behaviour, biology and ecology between generations of *Culicoides*.

Despite this apparent rapid adaptation to activity at cooler temperatures observed in populations in South East England, dominated by the subgenus *Avaritia*, testing of populations in a more northern latitude led to equivocal results. The high temperature threshold for activity in the NES cohort, of which > 99% of the total *Culicoides* were identified as C. *impunctatus*, was primarily driven by a limited flight response in populations tested within the bioassay. This was despite the fact that adults capable of flight towards light following trapping were pre-selected by the experimental design and conditions used were identical to the trials in South East England. The average flight activity response at the control temperature of 20 °C differed between cohorts; for total *Culicoides* collections across the SES and SEA cohorts, which were predominately comprised of *Culicoides* from the subgenus *Avaritia,* the average flight activity response at 20 °C was 86% and 78%, respectively. In contrast, a greatly reduced flight activity level of 20% was observed for total *Culicoides* collected in the NES cohort maintained at 20 °C, although this cohort was mainly comprised of *C. impunctatus*. *Culicoides* collected nearby in the SBS cohort had a greater species diversity and an increased flight activity at 20 °C of 29% suggesting that this was a species-specific response rather than a broader effect of sampling location. The reasons for the reduced response remain unclear but could represent a reduced phototactic response in particular species such as *C. impunctatus*.

The temperature thresholds for flight in populations dominated by the subgenus *Avaritia* populations in this study (4 °C in SEA and 10 °C in SES) were similar or slightly above those recorded for the temperate *C.* *oxystoma* in Japan (6 °C) [16], but were lower than the afrotropical *C. imicola* (14 °C) in RSA [15]. One major difference in experimental design between the current study and these was the avoidance of a sorting step that necessitated cold anaesthesia prior to testing activity. This was viewed as preferable to pre-exposing the *Culicoides* to cold, given previous evidence of cold hardening [34]. The experimental trade-off for avoiding this step was a lack of harmonisation of density in pots due to the difficulties of estimating numbers of *Culicoides* introduced. While this could have led to variation in density-dependent disturbance between flight activity pots, this was not evident in the study and would have been minimal in cooler temperatures where flight was restricted to a small proportion of the midges introduced. In addition, the requirement for vertical flight, rather than horizontal, enabled a more straightforward measure of activity, potentially reducing this factor.

In terms of wider policy implications of the study, the European Food Safety Authority (EFSA) has reported previously that, based on light-suction trapping the threshold temperature required to initiate *C.* *obsoletus* complex adult activity was 10 °C, with temperatures of ≤ 4 °C for longer than 10 days leading to an end to adult activity [35], although differentiation between survival and activity in this report is not well defined. The disparity between predictions of activity made from light-suction trapping and direct examination of flight could result from both the limits of using laboratory studies to define a response used in a field setting, and also the limits associated with trap surveillance. Light-suction traps are usually set at 1.5-2 m from ground level and have a limited effective range [26] and the period where the temperature range most often limits adult flight occurs during late autumn, winter and early spring, when risk of arbovirus incursion, and hence systematic trapping, is limited.

Vector surveillance programs, when limited, can underestimate the vector activity. The use of temperature-related baseline parameters to predict *Culicoides* activity, however, may provide an important tool when determining the optimal times to deploy surveillance trapping. This would provide a better assessment of transmission risk, particularly in the event of an incursion of an arbovirus. Surveillance programs using UV light suction traps can also actively select for host-seeking female *Culicoides* because of their increased attractiveness to light, therefore underestimating the number of *Culicoides* from the total population [36, 37] and also underestimating the levels of activity particularly of non-host seeking *Culicoides.* This study confirmed that there was no significant difference in the flight response towards a UV light stimulus between unpigmented and pigmented females of the subgenus *Avaritia* collected in either the summer or autumn from the same geographical location in the South East of England. This conclusion has implications when determining the SVFP, which is defined as < 5 parous (pigmented) *Culicoides* specifically caught in a UV light-suction trap collection over one night, despite both unpigmented and pigmented females being equally as active and both host seeking.

Whilst both collections from South East England consisted predominately of individuals belonging to the subgenus *Avaritia*, there was a far greater abundance of *C. scoticus* present in the SEA cohort (84%) compared with the SES cohort (37%). In this study we show, for the first time, that *C.* *scoticus* are significantly more active, under these laboratory conditions, at any temperature compared with *C. obsoletus* in both the summer and autumn, although this increased activity of *C.* *scoticus* could also have been influenced by differences in the species attractiveness to light. Due to difficulties separating these two species by morphological methods [38], differences in their behaviour as well as their vector competence have not yet been fully explored. Nevertheless, the differences in phototactic activity and seasonality between *C.* *obsoletus* and *C. scoticus* observed in this study could have implications for disease management, especially when determining the transmission season which could vary between species.

The flight activity of *Culicoides* adults can be influenced by several factors, but this present study has been limited to only temperature. Other environmental factors such as humidity, wind and rainfall have all been shown to be influential parameters in the flight response of *Culicoides* adults in the field [15, 39, 40]. Host-attraction cues may also play a role in the flight response of adult *Culicoides* populations. Consequently, measuring flight activity in response to temperature alone limits the ability to accurately define the multi-factorial flight response that would occur in the field. Furthermore, this study measured flight activity over a period of 24 h only, and so it is not known whether active individuals can remain active for > 24 h, particularly at low temperatures. Further investigations into the effect of prolonged exposure to cold temperatures on adult *Culicoides* activity are required to assess the effect of cold temperatures at the population level in northern Europe.

## Conclusions

The data presented here define cold temperature thresholds for flight activity in a range of UK *Culicoides* species and populations. Variation was observed in the subgenus *Avaritia* populations tested at different times of the year suggesting an effect of season on the activity of adult *Culicoides* whereby populations emerging later in the season possess a greater degree of cold tolerance. Further investigations are required to determine the effect of prolonged exposure to cold temperatures on *Culicoides* activity and survival to fully understand the consequence of cold winter temperatures on UK *Culicoides* populations and their ability to act as vectors of arboviruses.

## Supplementary information

**Additional file 1: Table S1.** Geographic and temporal differences between *Culicoides* cohorts used in the study.

**Additional file 2: Figure S1.** CDC miniature UV light-suction trap to collect live insects in the flight activity study.

**Additional file 3: Figure S2.** Methods used to collect and sort *Culicoides* collections for the flight activity study.

**Additional file 4: Table S2.** Differences observed in *Culicoides* populations used in each temperature trial across all cohorts tested.

**Additional file 5: Table S3.** Estimated coefficients (standard errors) in binomial family GLMMs for flight activity (proportion of midges flying) of *Culicoides* biting midges.

**Additional file 6: Table S4.** Estimated coefficients (standard errors) in binomial family GLMMs for flight activity (proportion of midges flying) of *Culicoides* biting midges at 12°C.

**Additional file 7: Table S5.** Estimated coefficients (standard errors) in binomial family GLMs for flight activity (proportion of midges flying) of *Culicoides obsoletus* and *Culicoides scoticus*.

## Data Availability

All data generated or analysed during this study are included in this published article and its additional files.

## Abbreviations

AHSV: African horse sickness virus
BTV: Bluetongue virus
CDC: Centers for Disease Control
EFSA: European Food Safety Authority
EIP: Extrinsic incubation period
GLMM: Generalised linear mixed model
GLM: Generalised linear model
IBH: Insect bite hypersensitivity
ITS: Internal transcribed spacer
NES: North East England summer cohort
PCR: Polymerase chain reaction
SBS: Scottish Borders summer cohort
SBV: Schmallenberg virus
SEA: South East England autumn cohort
SES: South East England summer cohort
SVFP: Seasonal vector-free period
UDG: Uracil-DNA-glycosylases
UV: Ultra-violet
