# Supplementary material for: Thermal limits for flight activity of field-collected Culicoides in the United Kingdom defined under laboratory conditions
Source: Parasit Vectors. 2021 Jan 18;14:55. doi: 10.1186/s13071-020-04552-x (PMC7814454; doi:10.1186/s13071-020-04552-x)
Supplement: Supplementary file 1 — Additional file 1: Table S1. Geographic and temporal differences between Culicoides cohorts used in the study. [file 13071_2020_4552_MOESM1_ESM.docx]

**Additional File 1**

**Table S1.** Geographic and temporal differences between *Culicoides* cohorts used in the study.

| **Cohort** | **Region** | **Time** | **Site  1*** | **Site 2*** | **Site  3*** | **Site  4*** | **Site  5*** |
| --- | --- | --- | --- | --- | --- | --- | --- |
| SES | SE England | June-August |  |  |  |  |  |
| SEA | SE England | September-October |  |  |  |  |  |
| NES | Kielder,  NE England | July |  |  |  |  |  |
| SBS | Scottish Borders | July |  |  |  |  |  |

*Sites used in each cohort are displayed by dark grey boxes, sites not used in each cohort are displayed by empty boxes.
