# Supplementary material for: Thermal limits for flight activity of field-collected Culicoides in the United Kingdom defined under laboratory conditions
Source: Parasit Vectors. 2021 Jan 18;14:55. doi: 10.1186/s13071-020-04552-x (PMC7814454; doi:10.1186/s13071-020-04552-x)
Supplement: Supplementary file 2 — Additional file 2: Figure S1. CDC miniature UV light-suction trap to collect live insects in the flight activity study. [file 13071_2020_4552_MOESM2_ESM.docx]

**Additional File 2**

**
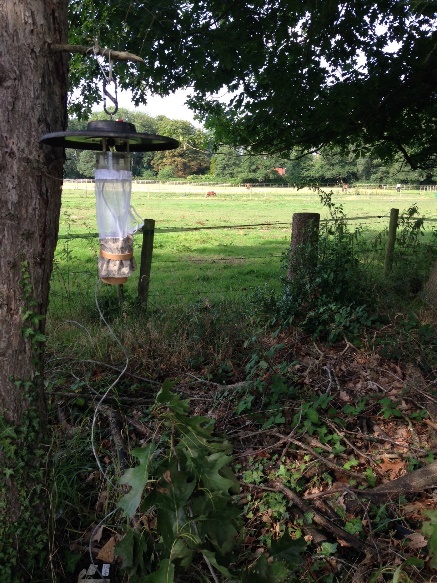
**

**Figure S1.** CDC miniature UV light-suction trap to collect live insects in the flight activity study.

The trap displayed here is located at site 2 in south east England. Collections were made into a 340ml cardboard collection cup containing a cotton pad soaked in 10% sucrose and paper towel cut into long, thin strips. Each trap was powered using a 12V lead acid sealed battery.
