# Supplementary material for: Thermal limits for flight activity of field-collected Culicoides in the United Kingdom defined under laboratory conditions
Source: Parasit Vectors. 2021 Jan 18;14:55. doi: 10.1186/s13071-020-04552-x (PMC7814454; doi:10.1186/s13071-020-04552-x)
Supplement: Supplementary file 3 — Additional file 3: Figure S2. Methods used to collect and sort Culicoides collections for the flight activity study. [file 13071_2020_4552_MOESM3_ESM.docx]

**Additional File 3**

1. CDC traps were set up two hours before sunset and run overnight to collect live insects into cardboard collection cups
2. Cardboard collection cups and attached mesh sleeve were collected within two hours of sunrise the following morning
3. Immediate transfer of cardboard collection cups and attached mesh sleeve from field sites to lab sites (The Pirbright Institute from sites 1-3 or Kielder Campsite from sites 4 and 5)
4. Two-hour acclimatisation from 20°C to test temperature (4°C to 14°C)
5. Live trap collections within 2 hours of sunrise
6. Cotton bung removed and pill box added at 0 hour. Pill boxes subsequently replaced at
   0.5, 1, 2, 4, 6, 8, 10, 24 hours
7. All insects from each pill box and the remainder of insects left in the flight activity pots were killed by freezing at -20°C and later counted and identified.
8. Live trap collections within 2 hours of sunrise
9. Sorting of live collections to collect approx. 150 active *Culicoides* into eight flight
   activity pots
10. Live trap collections within 2 hours of sunrise

**Figure S2.** Methods used to collect and sort *Culicoides* collections for the flight activity study.
