# Supplementary material for: Thermal limits for flight activity of field-collected Culicoides in the United Kingdom defined under laboratory conditions
Source: Parasit Vectors. 2021 Jan 18;14:55. doi: 10.1186/s13071-020-04552-x (PMC7814454; doi:10.1186/s13071-020-04552-x)
Supplement: Supplementary file 4 — Additional file 4: Table S2. Differences observed in Culicoides populations used in each temperature trial across all cohorts tested. [file 13071_2020_4552_MOESM4_ESM.docx]

**Additional File 4**

**Table S2.** Differences observed in *Culicoides* populations used in each temperature trial across all cohorts tested.

| Temperature  trial | Total number of *Culicoides* tested | Mean cumulative proportion of active *Culicoides* | Subgenus *Avaritia* | *C. achrayi* | *C. impunctatus* | Other *Culicoides* | Unpigmented | Pigmented | Gravid | Blood-fed | Male |
| --- | --- | --- | --- | --- | --- | --- | --- | --- | --- | --- | --- |
| **Cohort SES – south east England – June-August** | | | | | | | | | | | |
| 6°C | 941 | **0.4%** | 99.8% | 0.1% | 0.0% | 0.1% | 78.3% | 13.9% | 3.2% | 0.1% | 4.5% |
| 8°C | 455 | **0.6%** | 87.0% | 2.2% | 0.0% | 10.8% | 56.9% | 33.6% | 8.6% | 0.4% | 0.4% |
| 10°C | 1032 | **17.1%** | 99.5% | 0.0% | 0.0% | 0.5% | 82.6% | 14.7% | 1.5% | 1.1% | 0.2% |
| 12°C | 623 | **14.5%** | 92.0% | 5.0% | 0.0% | 3.0% | 52.6% | 38.4% | 7.9% | 0.2% | 1.0% |
| 14°C | 2535 | **72.3%** | 99.7% | 0.0% | 0.0% | 0.3% | 83.2% | 10.4% | 1.3% | 2.0% | 3.0% |
| **Cohort SEA – south east England – September-October** | | | | | | | | | | | |
| 2°C | 1363 | **0.2%** | 100.0% | 0.0% | 0.0% | 0.0% | 40.9% | 58.0% | 0.9% | 0.1% | 0.1% |
| 4°C | 1215 | **4.8%** | 99.4% | 0.0% | 0.0% | 0.6% | 43.1% | 55.4% | 0.7% | 0.7% | 0.1% |
| 6°C | 1061 | **19.3%** | 96.2% | 0.0% | 0.0% | 3.8% | 65.8% | 15.2% | 17.3% | 0.5% | 1.2% |
| 8°C | 1129 | **35.7%** | 99.9% | 0.0% | 0.0% | 0.1% | 71.4% | 28.0% | 0.3% | 0.2% | 0.2% |
| 10°C | 541 | **66.8%** | 99.6% | 0.0% | 0.0% | 0.4% | 76.5% | 19.8% | 1.1% | 2.2% | 0.4% |
| **Cohort NES – north east England - July** | | | | | | | | | | | |
| 8°C | 1812 | **0.0%** | 0.2% | 0.2% | 99.6% | 0.1% | 0.6% | 99.4% | 0.0% | 0.0% | 0.0% |
| 10°C | 1562 | **0.8%** | 0.0% | 0.1% | 99.7% | 0.2% | 0.5% | 99.2% | 0.2% | 0.0% | 0.1% |
| 12°C | 1864 | **1.7%** | 0.1% | 0.5% | 99.1% | 0.3% | 1.3% | 97.9% | 0.5% | 0.0% | 0.3% |
| 14°C | 1405 | **9.1%** | 0.0% | 0.1% | 99.8% | 0.1% | 0.8% | 99.1% | 0.0% | 0.0% | 0.1% |
| **Cohort SBS – Scottish Borders - July** | | | | | | | | | | | |
| 12°C | 585 | **14.4%** | 29.7% | 35.9% | 29.1% | 5.3% | 37.9% | 44.8% | 3.2% | 0.2% | 13.8% |
