# Supplementary material for: Thermal limits for flight activity of field-collected Culicoides in the United Kingdom defined under laboratory conditions
Source: Parasit Vectors. 2021 Jan 18;14:55. doi: 10.1186/s13071-020-04552-x (PMC7814454; doi:10.1186/s13071-020-04552-x)
Supplement: Supplementary file 5 — Additional file 5: Table S3. Estimated coefficients (standard errors) in binomial family GLMMs for flight activity (proportion of midges flying) of Culicoides biting midges. [file 13071_2020_4552_MOESM5_ESM.docx]

**Additional File 5**

**Table S3.** Estimated coefficients (standard errors) in binomial family GLMMs for flight activity (proportion of midges flying) of *Culicoides* biting midges.

| parameter | all midges | unpigmented females | pigmented females |
| --- | --- | --- | --- |
| total *Culicoides* |  |  |  |
| intercept | -11.00 (0.63) | -10.66 (0.77) | -11.51 (0.79) |
| temperature | 0.84 (0.05) | 0.83 (0.07) | 0.86 (0.06) |
| cohort |  |  |  |
| SES | baseline | baseline | baseline |
| SEA | 3.88 (0.37) | 3.59 (0.42) | 4.46 (0.46) |
| NES | -3.20 (0.33) | - | -2.99 (0.34) |
| random effect (pot)† | 0.93 | 1.04 | 0.89 |
| *Avaritia* subgenus |  |  |  |
| intercept | -10.95 (0.71) | -10.06 (0.76) | -11.62 (0.94) |
| temperature | 0.84 (0.06) | 0.83 (0.07) | 0.87 (0.07) |
| cohort |  |  |  |
| SES | baseline | baseline | baseline |
| SEA | 3.83 (0.41) | 3.56 (0.42) | 4.51 (0.54) |
| random effect (pot)† | 1.02 | 1.03 | 1.01 |
| *Culicoides impuncatatus*‡ |  |  |  |
| intercept | -14.08 (1.23) | - | -13.95 (1.17) |
| temperature | 0.84 (0.09) | - | 0.83 (0.09) |
| random effect (pot)† | 0.41 | - | 0.37 |

† standard deviation (on logit scale)

‡ NES cohort only
