# Supplementary material for: Thermal limits for flight activity of field-collected Culicoides in the United Kingdom defined under laboratory conditions
Source: Parasit Vectors. 2021 Jan 18;14:55. doi: 10.1186/s13071-020-04552-x (PMC7814454; doi:10.1186/s13071-020-04552-x)
Supplement: Supplementary file 6 — Additional file 6: Table S4. Estimated coefficients (standard errors) in binomial family GLMMs for flight activity (proportion of midges flying) of Culicoides biting midges at 12°C. [file 13071_2020_4552_MOESM6_ESM.docx]

**Additional File 6**

**Table S4.** Estimated coefficients (standard errors) in binomial family GLMMs for flight activity (proportion of midges flying) of *Culicoides* biting midges at 12°C.

| parameter | all midges | unpigmented females | pigmented females |
| --- | --- | --- | --- |
| total *Culicoides* |  |  |  |
| intercept | -1.85 (0.26) | -1.53 (0.32) | -2.08 (0.28) |
| cohort |  |  |  |
| SES | baseline | baseline | baseline |
| NES | -2.26 (0.38) | - | -2.05 (0.37) |
| SBS | 0.03 (0.36) | 0.35 (0.44) | -0.30 (0.39) |
| random effect (pot)† | 0.57 | 0.62 | 0.38 |
| *Avaritia* subgenus |  |  |  |
| intercept | -1.71 (0.28) | -1.41 (0.35) | -2.01 (0.31) |
| cohort |  |  |  |
| SES | baseline | baseline | baseline |
| SBS | 0.35 (0.42) | 0.10 (0.56) | 1.44 (0.53) |
| random effect (pot)† | 0.52 | 0.69 | 0.44 |
| *Culicoides impuncatatus* |  |  |  |
| intercept | -4.20 (0.29) | - | - |
| cohort |  |  |  |
| NES | baseline | - | - |
| SBS | -1.01 (1.05) | - | - |
| random effect (pot)† | 0.42 | - | - |

† standard deviation (on logit scale)
