# Supplementary material for: Thermal limits for flight activity of field-collected Culicoides in the United Kingdom defined under laboratory conditions
Source: Parasit Vectors. 2021 Jan 18;14:55. doi: 10.1186/s13071-020-04552-x (PMC7814454; doi:10.1186/s13071-020-04552-x)
Supplement: Supplementary file 7 — Additional file 7: Table S5. Estimated coefficients (standard errors) in binomial family GLMs for flight activity (proportion of midges flying) of Culicoides obsoletus and Culicoides scoticus. [file 13071_2020_4552_MOESM7_ESM.docx]

**Additional File 7**

**Table S5.** Estimated coefficients (standard errors) in binomial family GLMs for flight activity (proportion of midges flying) of *Culicoides obsoletus* and *Culicoides scoticus*.

| parameter | all midges | unpigmented females | pigmented females |
| --- | --- | --- | --- |
| intercept | -14.11 (1.08) | -15.25 (1.32) | -18.81 (5.06) |
| temperature | 1.24 (0.09) | 1.33 (0.11) | 1.76 (0.49) |
| cohort |  |  |  |
| SES | baseline | baseline | baseline |
| SEA | 8.51 (1.20) | 11.21 (1.38) | 10.70 (5.21) |
| species |  |  |  |
| *C. obsoletus* | baseline | baseline | baseline |
| *C. scoticus* | 0.25 (0.32) | 0.61 (0.13) | -1.36 (1.16) |
| temperature:cohort |  |  |  |
| SES | baseline | baseline | baseline |
| SEA | -0.76 (0.10) | -0.99 (0.13) | -0.94 (0.50) |
| cohort:species |  |  |  |
| SEA, *C. scoticus* | 0.99 (0.46) | - | 2.98 (1.40) |
